# Cardiac Magnetic Resonance to Define Myocardial Structure in Obesity-Associated Heart Failure

**DOI:** 10.1007/s11886-026-02372-6

**Published:** 2026-04-22

**Authors:** Jamey A. Cutts, Frederick H. Epstein, Amit R. Patel

**Affiliations:** 1 Department of Medicine, Division of Cardiovascular Medicine, University of Virginia, Charlottesville, VA USA; 2 Department of Biomedical Engineering, University of Virginia, Charlottesville, VA USA

**Keywords:** Cardiac MRI, Obesity, HFpEF, Heart failure

## Abstract

**Purpose of Review:**

The global prevalence of both obesity and heart failure continues to rise, and accumulating evidence suggests that their association is likely causal, giving rise to a distinct heart failure phenotype with unique pathophysiologic features compared to non-obese individuals. This review highlights the evolving role of cardiac magnetic resonance imaging (CMR) in the assessment and management of obesity-related heart failure, emphasizing it’s ability to characterize the structural, functional, and tissue-level cardiovascular abnormalities that define this increasingly prevalent condition.

**Recent Findings:**

Obesity plays a central role in the development of heart failure with preserved ejection fraction (HFpEF), contributing to a unique pathophysiologic cardiovascular phenotype through mechanisms such as myocardial inflammation, diffuse fibrosis, abnormal ventricular loading, and pathologic expansion of adjacent epicardial adipose tissue. These structural and physiologic changes in turn lead to disproportionate atrial and ventricular remodeling, pronounced diastolic dysfunction, cardiac microvascular dysfunction, impaired interventricular mechanics, and elevated cardiac filling pressures. Together, these alterations contribute to the heightened symptom burden, exercise intolerance, and adverse outcomes observed in obese patients with HFpEF. Cardiac magnetic resonance imaging (CMR) provides a non-invasive, comprehensive platform to assess these hemodynamic, structural, and tissue-level abnormalities through an ever-expanding suite of quantitative imaging tools.

**Summary:**

CMR, with its high spatial resolution, advanced tissue characterization, and comprehensive evaluation of cardiac structure and function, has emerged as a pivotal modality for the diagnosis, phenotyping, and risk stratification of obesity related HFpEF, while also enabling differentiation from important phenocopies and alternative causes of heart failure.

**Graphical Abstract:**

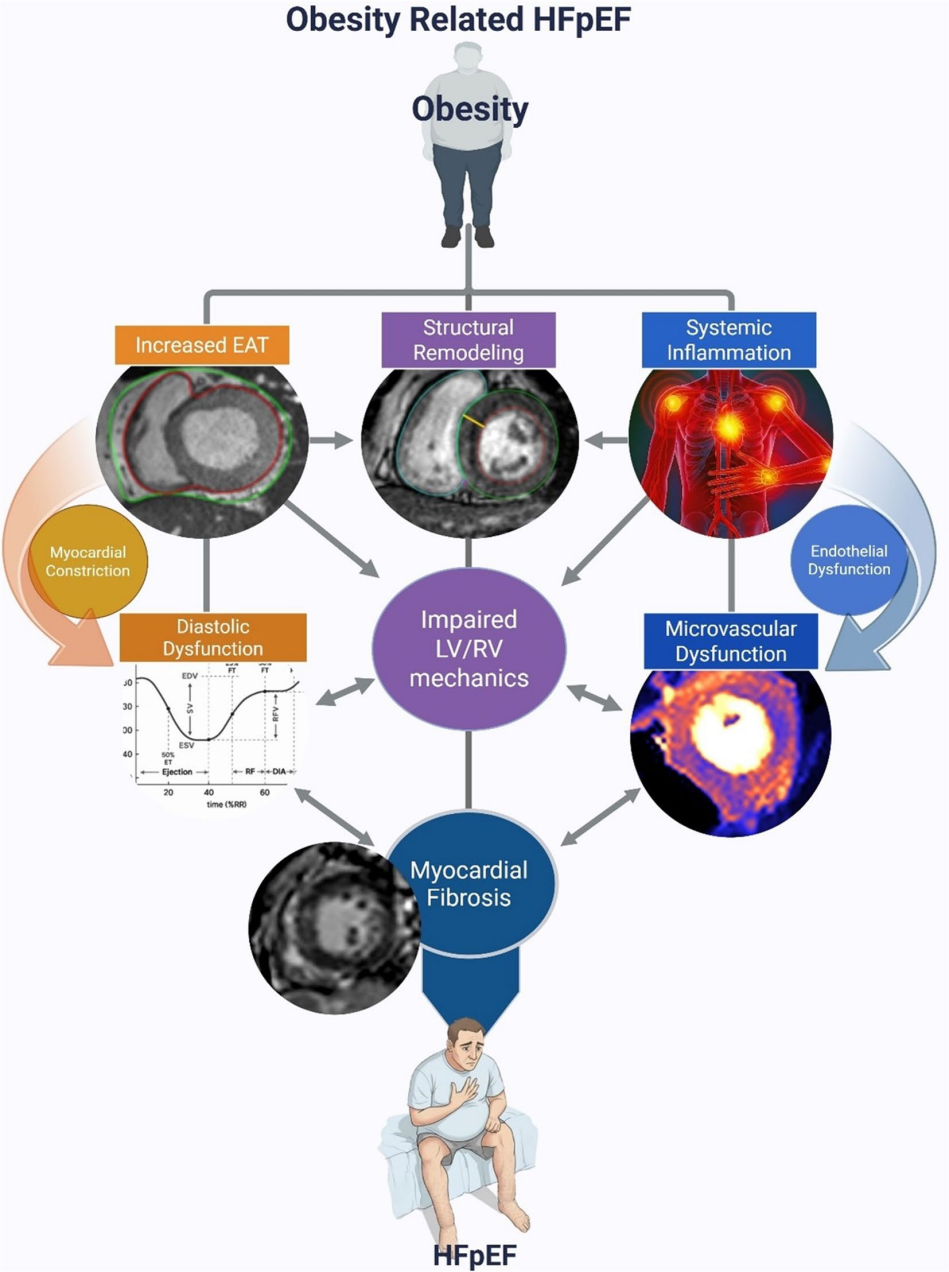

## Introduction

Heart failure (HF) remains a leading cause of morbidity and mortality worldwide with heart failure with preserved ejection fraction (HFpEF) now comprising the majority of new heart failure cases diagnosed annually [1]. This rise in HFpEF prevalence is at least partly, if not more significantly, due to the parallel rise in obesity. Recent estimates indicate that around 40% of adults in the United States and over 80% of HFpEF patients qualify as overweight or obese by traditional BMI standards. When evaluating central adiposity, a better surrogate of visceral adiposity than BMI, 96% of patients with HFpEF had elevated waist-to-height ratios which correlated linearly with total HF events [2, 3]. Epidemiologic studies have also consistently demonstrated a strong association not only between obesity and incident heart failure but also with higher symptom burden in obese patients with HFpEF. Despite the increased burden of symptoms, obese patients often present with normal or near normal resting filling pressures and lower biomarker levels compared to their non-obese counterparts. As a result, understanding the cardiovascular consequences of obesity has become a growing area of interest, particularly in the context of obesity related heart failure.

HFpEF has been classically accepted as a disease process driven by age-related myocardial stiffening, hypertensive remodeling, and microvascular dysfunction that promote diastolic dysfunction and impaired relaxation. However, more recently there has been emerging data that these mechanisms while still involved, may be less important in obesity related HFpEF which is increasingly recognized as a distinct clinical phenotype characterized by systemic and cardiac alterations driven by excess adiposity. The American Heart Association and American College of Cardiology both highlight that in these patients, accumulation of visceral and epicardial adipose tissue leads to low-grade systemic inflammation, myocardial fibrosis, and pericardial constraint, which together contribute to impaired diastolic filling and increased ventricular stiffness [1, 4–6]. Obese individuals with HFpEF also tend to be younger and have elevated circulating plasma volume, higher resting cardiac output, and increased arterial stiffness resulting in a unique hemodynamic profile that can mask the severity of HF symptoms at rest but may cause pronounced exertional intolerance [4, 7]. Imaging and biomarker studies have further demonstrated that obesity related HFpEF is associated with distinct alterations in myocardial structure and function, such as increased left ventricular mass and concentric remodeling, even in the absence of traditional risk factors [7]. The differences in underlying pathophysiology between obese and non-obese HFpEF not only affect clinical presentation and disease trajectory but more importantly may influence responses to therapy, highlighting the importance of phenotyping HFpEF patients to enable more tailored and effective management strategies.

Cardiac magnetic resonance imaging (CMR) offers a highly reproducible method for assessing many of the above-mentioned changes in the cardiac structure associated with obesity related HFpEF. It can also evaluate intrinsic characteristics of tissue using techniques like parametric mapping, myocardial blood flow assessment, and late gadolinium enhancement that cannot be accomplished by most other non-invasive modalities. These make it particularly well suited to detect changes such as interstitial fibrosis, microvascular dysfunction, geometric remodeling, and inflammation among others. As there is no ionizing radiation and newer macrocyclic gadolinium contrast agents are safe even in end stage renal disease, it is also well suited for serial assessments with minimal risk to patients. For these reasons, CMR plays a pivotal role in obesity related heart failure enabling more precise phenotyping and potentially informing targeted therapeutic strategies in the future. In this review, we summarize the current role for CMR in obesity related heart failure.

## Body

### Structural Changes in Obesity Related HFpEF

**Fig. 1:**
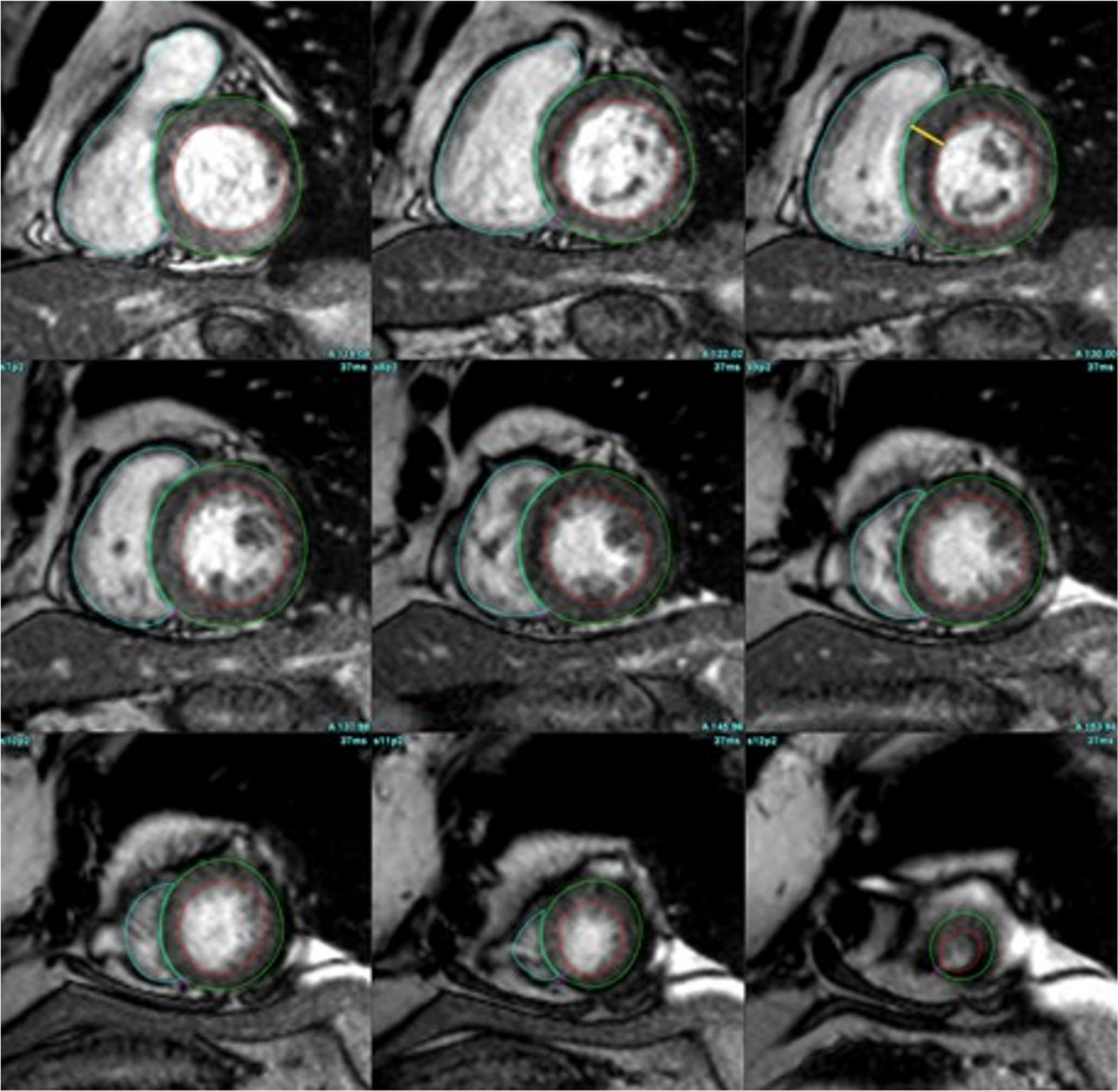
SSFP cine short axis stack with endocardial and epicardial contours to calculate LV mass and LV volume. SSFP – steady state free procession. LV – left ventricle

Obesity imposes a chronic hemodynamic and metabolic burden on the heart, contributing to several key changes in the cardiac structure that contribute to the development of HF. Left ventricular (LV) remodeling is one of the prominent changes in obese related HFpEF. Driven by elevated circulating plasma volume and higher cardiac output - which can be almost 2 L/min greater than that in lean patients - LV dilation manifests as an increase in the LV end-diastolic volume (LVEDV) [8]. Obese patients with HFpEF had higher LVEDV of 133 mL compared to just 97 mL in non-obese patients with HFpEF [9]. This cavity dilation is followed by a compensatory increase in LV wall thickness and thus LV mass which is driven primarily by increased aortic stiffness, systemic hypertension, and adiposity related neurohormonal signaling. LV mass has been shown to be as high as 40 g higher in obese vs. non-obese individuals [8]. This eventually leads to an elevated LV mass to volume ratio also known as concentric remodeling. This remodeling has been linked to reduced cardiorespiratory fitness - a stronger predictor of adverse cardiovascular outcomes than many traditional risk factors [5, 10]. CMR using steady state free procession (SSFP) sequences can image the cardiac chambers in all planes with high diagnostic accuracy which allows for direct measurement of the LV mass, wall thickness, and chamber sizes eliminating the need for geometric assumptions as is required with echocardiography as shown in Fig. 1. It is also highly reproducible allowing for serial assessments for disease progression and therapy efficacy. Recently, twelve months of tirzepatide therapy was shown to result in an 11-gram reduction in LV mass relative to placebo and a trend toward improvement in LVEDV. This may provide a possible explanation for the reduction in cardiac events noted in the Tirzepatide for Heart Failure with Preserved Ejection Fraction study [11, 12].

**Fig. 2:**
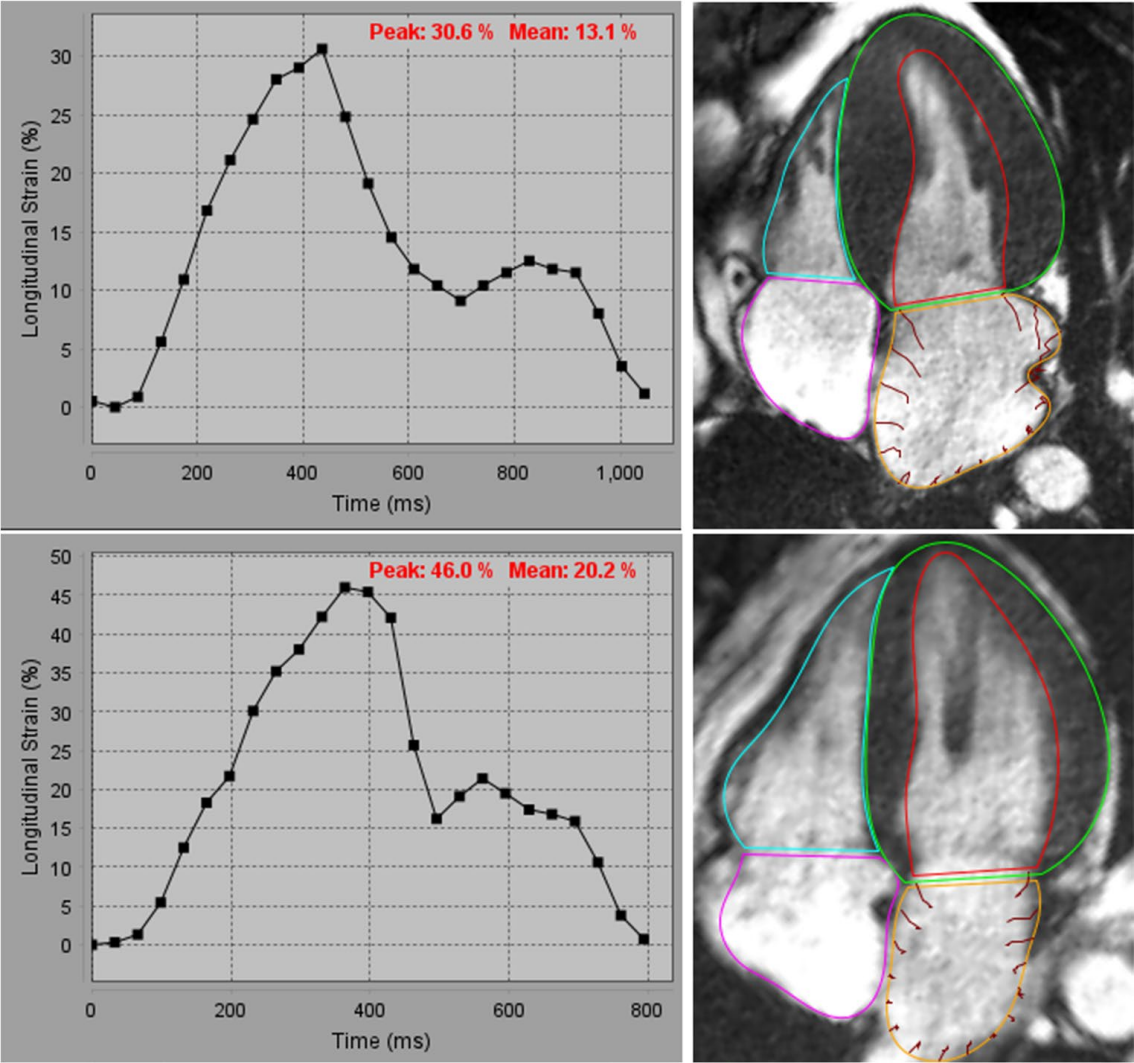
Top left: Reduced left atrial strain in a patient with HFpEF. Top right: Corresponding four chamber long axis cine with LA segmentation in orange. Bottom left: Control patient with normal left atrial strain curve. Bottom right: SSFP four chamber long axis sequence with orange countour around the left atrium used to generate the preceding strain curve. HFpEF – heart failure with perserved ejection fraction. LA – left atrial. SSFP – steady state free procession

In response to the chronically elevated filling pressures, the left atrium (LA) also undergoes changes that are reliably assessed by CMR. While left atrial enlargement is a hallmark of all comers with HFpEF due to chronic diastolic dysfunction and elevated LV filling pressures it is even more prevalent in obesity related HFpEF due to additional metabolic stressors such as obstructive sleep apnea, systemic inflammation, and accumulation of epicardial adipose tissue [6, 7, 13]. Unlike echocardiography, which may be limited by acoustic windows, CMR provides complete visualization of the left atrium, enabling precise assessment of the atrial volume and atrial function allowing for the generation of time-volume curves (see Fig. 2). Feature tracking strain can assess LA function with high accuracy in all three phases of atrial strain – reservoir, conduit, and booster and may precede overt changes in atrial size [14–16].

The right ventricle (RV) is also affected by the chronic effects of obesity and consequently undergoes structural and functional changes. Typically, RV dilation and dysfunction occur after chronic exposure to elevated left atrial pressures from LV diastolic dysfunction. Compared to non-obese counterparts, obese patients with HFpEF exhibited more diastolic dysfunction and larger RV end-diastolic and end -systolic volumes that correlated with body mass [4]. Additionally, obesity has been associated with significant reductions in RV ejection fraction (RVEF) and impaired myocardial energy utilization – both of which likely contribute to the increased symptom burden in this population. In addition to resting abnormalities, obese patients also demonstrate decreased RV systolic contractile reserve during dobutamine stress CMR which correlates with heightened exertional intolerance [17]. Notably, RV dilation and RV ejection fraction have been shown to improve following weight loss, suggesting this obesity related RV dysfunction is at least partially reversible [17].

**Fig. 3:**
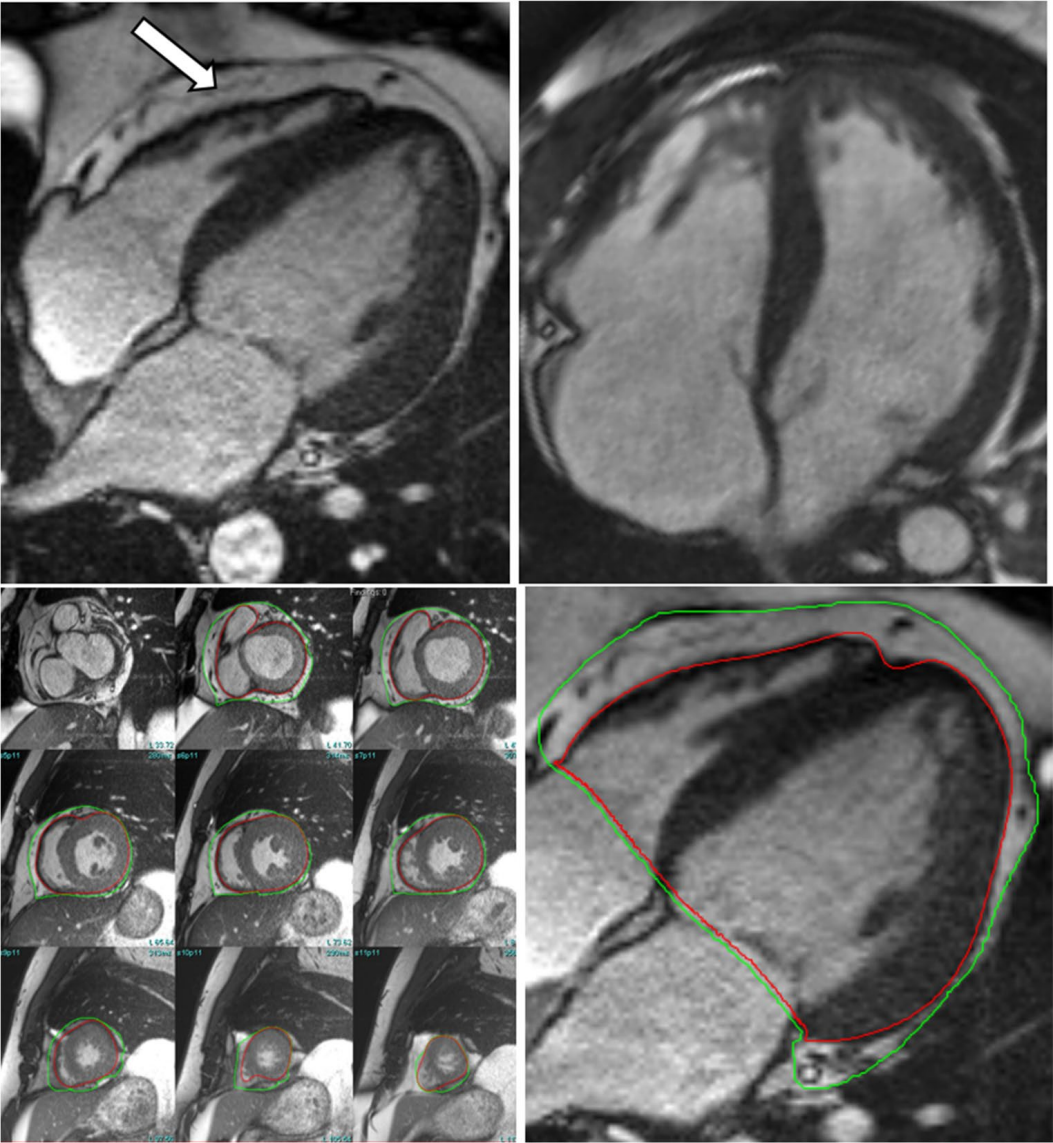
Top left: Four chamber SSFP cine image showing increased EAT (white arrow). Top right: Four chamber SSFP cine with normal EAT volume. The bottom row shows different methods for quantifying EAT. Bottom left: Short axis SSFP stack with EAT segmented between the red (external surface of myocardium) and green (visceral pericardium) contours. Bottom right: Long axis four chamber SSFP image segmenting the epicardial adipose tissue. SSFP – steady state free procession. EAT – epicardial adipose tissue

Another key structural alteration in obese related HFpEF is expansion of epicardial adipose tissue (EAT), the visceral fat located between the myocardium and the visceral pericardium. Under the systemic inflammatory conditions associated with obesity, EAT likely undergoes pathologic transformation, contributing to heart failure through several mechanisms. These include direct mechanical restriction of the adjacent myocardium and secondary myocardial effects via proinflammatory paracrine signaling. CMR is a highly effective tool for assessing EAT, allowing for precise quantification of EAT volume which tends to be increased in obese patients with HFpEF (see Fig. 3) [6, 13, 18]. It has been shown that individuals with increased EAT volume have higher filling pressures and greater pericardial constraint at rest and with exercise than non-obese counterparts which may explain their reduced exercise capacity [19]. CMR also enables direct assessment of the tissue characteristics of EAT. Emerging techniques such as fatty acid composition (FAC) allow quantification of the relative percentages of saturated and unsaturated fatty acids within EAT. In preclinical models, these correlated with histologic and cytokine markers of inflammation, suggesting that epicardial adipose tissue fatty acid composition (EAT FAC) EAT FAC imaging may help identify proinflammatory EAT phenotypes [20]. Preclinical models have also shown that EAT volume and EAT FAC can be improved with empagliflozin therapy which may further expand our understanding of the benefits of sodium-glucose co-transporter 2 inhibitor therapy in HFpEF [21]. GLP-1 receptor agonists are also being investigated. It has recently been shown that Tirzepatide therapy resulted in a statistically significant placebo corrected reduction in paracardiac adipose tissue (the sum of EAT and the fat directly outside of the pericardium) of approximately 20%, although this effect was primarily driven by reductions in the extra-pericardial adipose tissue [12, 22, 23].

### Diastolic Dysfunction

Diastolic dysfunction is a central pathophysiologic feature of HFpEF. The concentric remodeling, elevated filling pressures, myocardial stiffness, and chronic systemic inflammation mentioned above all contribute to impaired LV relaxation and compliance which tends to be even more prominent during exercise for obese patients with HFpEF [24]. While echocardiography remains the first line modality for the non-invasive evaluation of diastolic dysfunction, diastolic assessment by CMR continues to evolve. Time volume curves quantify changes in LV volume over time by segmenting the myocardium in short axis SSFP cine images which can then be plotted on a standard grid using LV volume along the Y axis and time (usually as a percentage of the R-R interval) on the X axis (see image below). This myocardial contouring is done for every phase of the cardiac cycle enabling assessment of early (E) and late (A) filling rates, E/A ratio, peak filling rate (PFR), and time to peak filling which can be used to evaluate impaired relaxation and reduced compliance and have been shown to correlate with echocardiographic diastolic dysfunction [24]. CMR has also been used to predict elevated pulmonary capillary wedge pressure (PCWP). Multivariate regression analysis was used to determine the common CMR measurements independently associated with elevated PCWP obtained by RHC. Two of these variables – LA volume and LV Mass – were then used to derive a formula from which CMR derived PCWP can be calculated. This CMR derived PCWP outperformed TTE at classifying patients with either normal or elevated filling pressures when compared to the gold standard invasive assessment. An elevated CMR derived PCWP was also an independent predictor of survival [25]. While pharmacologic stress CMR is a well-established tool for assessing ischemia, emerging research is exploring the use of exercise CMR using MRI compatible recumbent bikes as well. This is particularly relevant in obese related HFpEF as more than half of patients exhibit normal filling pressures at rest, making assessment of cardiac function during exercise crucial for diagnosis. Although exercise CMR has historically been limited by breath-hold requirements and motion artifact, advances in real time imaging techniques have been shown to be to be an accurate and feasible non-invasive method for identifying features of HFpEF under physiologic stress [26].

### Coronary Microvascular Dysfunction

**Fig. 4:**
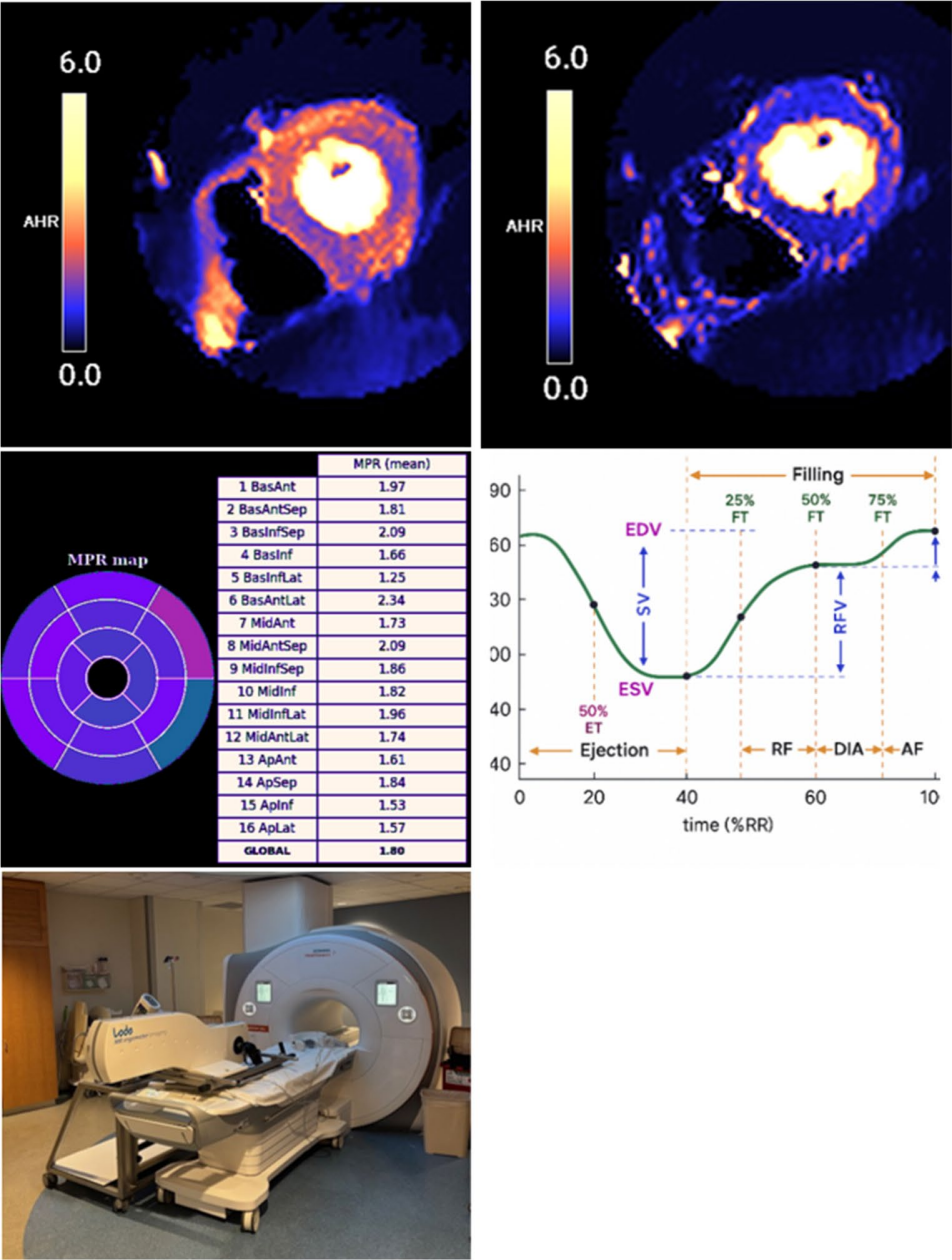
Top left: color map showing rest perfusion sequence with normal perfusion. Top right: color map showing globally reduced stress myocardial blood flows in a HFpEF patient. Middle left: globally reduced MPR of 1.8 consistent with CMD. Middle right: time volume curve tracking ventricular filling throughout the cardiac cycle. (From Goyal N et al. [39], with permission from Elsevier.) Bottom: MRI compatible exercise bike for stress CMR. CMD – coronary microvascular disease. HFpEF – heart failure with preserved ejection fraction. MPR – myocardial perfusion reserve. CMR – cardiac magnetic resonance

Another key feature of obesity related HFpEF is coronary microvascular disease (CMD) which has become increasingly recognized as a contributor to the pathophysiology of HFpEF and is present in over 70% of patients with HFpEF. In obesity, systemic inflammation, insulin resistance, and adipokine imbalance contribute to endothelial dysfunction and impaired coronary vasodilator reserve [27, 28]. These changes compromise myocardial perfusion even in the absence of obstructive epicardial coronary artery disease. Stress CMR offers a method to quantitate resting and stress myocardial blood flow utilizing gadolinium-based contrast and a vasodilator stress agent. These MBFs can be used to derive the myocardial perfusion reserve (MPR) - also referred to in literature as coronary flow reserve and myocardial flow reserve – both globally and by vascular territory allowing for the detection of global or regional CMD (see Fig. 4). In obese individuals, reduced MPR has been associated with greater symptom burden, impaired exercise capacity, and worse clinical outcomes [28]. Oxygen sensitive CMR (OS-CMR) is a novel technique using hyperventilation followed by a breath hold to evaluate myocardial oxygenation reserve, a marker of function of the coronary vasculature, without the need for exogenous contrast. An abnormal myocardial oxygenation reserve by OS-CMR was found in 96% of patients with HFpEF and correlated with myocardial edema, wall thickness, and diastolic dysfunction [29]. This technique may provide an additional method to evaluate for microvascular abnormalities seen in HFpEF without the need for a contrast agent.

### Tissue Characterization

A unique and important strength of CMR is the ability to directly assess myocardial tissue composition through the use of late gadolinium enhancement (LGE) and parametric T1 and T2 mapping techniques. Diffuse interstitial fibrosis has been implicated as one potential driver of diastolic dysfunction and LV stiffness in HFpEF and can be evaluated with T1 mapping sequences. This sensitive technique allows for the detection of diffuse fibrosis even in the absence of late gadolinium enhancement (LGE). In addition, T1 mapping can be used to derive the extracellular volume (ECV), a marker of the percentage of myocardium not occupied by myocytes. Both native T1 and ECV have been shown to be elevated in obesity related HFpEF compared to healthy controls [30–32]. Elevated ECV has also been associated with increased LV stiffness which may explain the greater symptom burden, reduced exercise capacity, and elevated risk of hospitalization for heart failure observed in this population [28, 33]. The value of T2 mapping in HFpEF is not yet well defined. T2 values, a marker of myocardial edema, have been well validated as a marker of acuity and inflammation of the myocardium. While there is evidence that T2 can be elevated during acute decompensations of heart failure its use remains fairly limited in HFpEF as a diagnostic or prognostic marker. Elevations in T2 (outside of a patient with acutely decompensated HF) should prompt consideration of alternative cardiomyopathy such as myocarditis or sarcoidosis rather than conventional HFpEF [34]. MR spectroscopy, another tool of uncertain significance in HFpEF currently due to its long acquisition times and difficult technical considerations, may garner a larger role in the future as it allows for a non-invasive assessment of myocardial triglyceride (TG) content that correlates well with endomyocardial biopsy. Elevations in the myocardial TG level have also been linked to diastolic dysfunction [35].

Late gadolinium enhancement is a technique that detects focal myocardial fibrosis or scarring by identifying regions of delayed washout of gadolinium contrast relative to normal myocardium. LGE has been shown to be a poor prognostic marker in many pathologic cardiac conditions including HFpEF where it was present in 36% of patients and correlated with higher risk of heart failure hospitalization and death [36]. There is no pathognomonic LGE pattern in HFpEF; however, when present it tends to appear in a mid-myocardial or subepicardial distribution and may range from focal to more diffuse involvement. Although less common than in HFrEF, the identification of LGE in HFpEF identifies a higher risk subgroup with more advanced structural remodeling and poorer prognosis. Additionally, certain LGE patterns may suggest alternative or coexisting pathologies that could be contributing to the patient’s symptoms.

### HFpEF Mimics

**Fig. 5:**
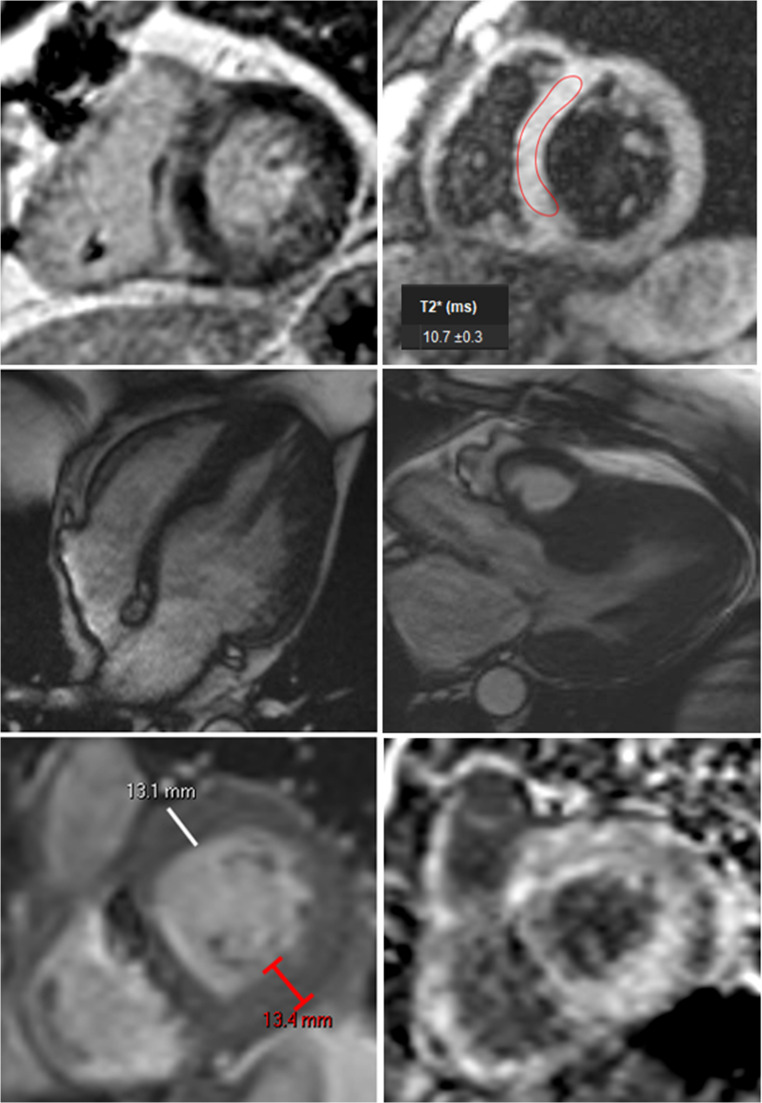
Top left: Short axis LGE image from a patient with cardiac sarcoidosis showing mid-wall LGE hooking from the LV into the RV myocardium. Top right: Short axis T2* map with moderate to severely reduced T2* value consistent with cardiac hemochromatosis. Middle left: Four chamber long axis SSFP with apical HCM. Middle right: Three chamber long axis SSFP with asymmetric septal hypertrophy and SAM of anterior mitral leaflet consistent with obstructive hypertrophic cardiomyopathy. Bottom left: Moderate concentric hypertrophy in a patient with cardiac amyloidosis. Bottom right: Diffuse subendocardial LGE from the prior patient with cardiac amyloidosis. LGE – late gadolinium enhancement. LV – left ventricle. RV – right ventricle. SSFP – steady state free procession. HCM – hypertrophic cardiomyopathy. SAM – systolic anterior motion

Another important role for CMR in the diagnosis and management of HFpEF is the identification or exclusion of HFpEF mimics that may require alternative, disease-specific therapies. These include inflammatory, infiltrative, and restrictive conditions such as cardiac amyloidosis, hypertrophic cardiomyopathy, cardiac sarcoidosis, pericardial constriction, myocardial siderosis, and glycogen storage disorders amongst others (see Fig. 5). Prior studies have shown that 27% of patients with suspected HFpEF who underwent CMR were found to have previously unrecognized alternative pathology [37, 38]. CMR provides additional insight into the potential presence of these alternative entities. Given the therapeutic and prognostic implications, CMR should be considered in all patients with known or suspected HFpEF who have atypical risk factors such as severe or asymmetric left ventricular hypertrophy (LVH), LVH in the absence of longstanding hypertension, low QRS voltage, carpal tunnel syndrome, left ventricular outflow tract obstruction, conduction system disease, or history concerning for prior pericarditis amongst others.

## Conclusion

Obesity related HFpEF represents a distinct and increasingly prevalent phenotype of heart failure, characterized by unique structural, functional, and hemodynamic alterations both at rest and with exertion. Cardiac MRI plays a central role in its evaluation, offering high diagnostic accuracy in detecting the concentric remodeling, atrial enlargement, myocardial fibrosis, and microvascular dysfunction commonly seen in this population. In addition to improving diagnostic precision, CMR enhances risk stratification and helps identify alternative or coexisting pathologies that may mimic HFpEF. CMR should be considered an essential tool in the diagnostic and management algorithm for patients with suspected or confirmed HFpEF.

While these advanced CMR techniques hold promise for improving understanding of the pathophysiologic changes underlying obesity related HFpEF as well as enhancing its clinical evaluation and management there are several important practical considerations that must also be considered. Although access to CMR is increasing, availability remains limited in some areas, particularly for some of the more advanced sequences and techniques discussed in this paper. For more widespread adoption in the future, standardization of protocol acquisition and analysis and reduction of scan times through development of faster sequences will be important. Machine learning and artificial intelligence may help facilitate these advances. At the same time, patient related factors such as body habitus, the ability to lie flat and perform breath holds, and cost must also be considered. Addressing these challenges will be essential for successful widespread implementation of CMR in the evaluation of obesity related HFpEF.

## Key References

- Kittleson Michelle M., Panjrath Gurusher S., Amancherla Kaushik, et al. 2023 ACC Expert Consensus Decision Pathway on Management of Heart Failure With Preserved Ejection Fraction. *JACC*. 2023;81(18):1835–1878. 10.1016/j.jacc.2023.03.393
  - ○ This expert consensus pathway establishes a framework for the diagnosis and management of heart failure with preserved ejection fraction including important considerations for the role of imaging in these patients.
- Baron T, Gerovasileiou S, Flachskampf FA. The role of imaging in the selection of patients for HFpEF therapy. *Eur Heart J - Cardiovasc Imaging*. 2023;24(10):1343–1351. 10.1093/ehjci/jead137
  - ○ This paper highlights many of the important roles for cardiac MRI in the diagnosis of HFpEF and exclusion of important comorbid conditions.
- Ipek R, Holland J, Cramer M, Rider O. CMR to characterize myocardial structure and function in heart failure with preserved left ventricular ejection fraction. Eur Heart J - Cardiovasc Imaging. 2024;25(11):1491–1504. 10.1093/ehjci/jeae224
  - ○ This paper provides a comprehensive state-of-the-art overview of how multiparametric CMR characterizes myocardial structure, function, and tissue composition in HFpEF.

## Data Availability

No datasets were generated or analysed during the current study.

## Abbreviations

CMR: Cardiac Magnetic Resonance
CMD: Coronary Microvascular Disease
EAT: Epicardial Adipose Tissue
ECV: Extracellular Volume
EAT FAC: Epicardial Adipose Tissue Fatty Acid Composition
FAC: Fatty Acid Composition
HF: Heart Failure
HFpEF: Heart Failure with Preserved Ejection Fraction
HFrEF: Heart Failure with Reduced Ejection Fraction
LGE: Late gadolinium enhancement
LA: Left atrium
LV: Left Ventricle
LVEDV: Left Ventricular End Diastolic Volume
MPR: Myocardial Perfusion Reserve
OS-CMR: Oxygen Sensitive Cardiac Magnetic Resonance
PFR: Peak Filling Rate
PCWP: Pulmonary Capillary Wedge Pressure
RV: Right Ventricle
RVEF: Right Ventricular Ejection Fraction
SSFP: Steady State Free Procession
TTE: Transthoracic echo
